# A review and meta-analysis of stem cell therapies in stroke patients: effectiveness and safety evaluation

**DOI:** 10.1007/s10072-023-07032-z

**Published:** 2023-09-21

**Authors:** L. Hovhannisyan, S. Khachatryan, A. Khamperyan, S. Matinyan

**Affiliations:** 1 MatinyanLab Foundation, 0096 Yerevan, Armenia; 2 Department of Radiation Oncology, Inselspital, Bern University Hospital, University of Bern, 3008 Bern, Switzerland; 3 Faculty of Science, University of Basel, Basel, Switzerland

**Keywords:** Stroke, Stem cell, NIHSS, mRS, BI

## Abstract

**Purpose:**

Stem cells have been extensively used during the last decade to improve clinical outcomes after stroke. The dramatic increase in trials in this field has led us to perform a systematic review and meta-analysis to understand the safety, effectiveness, and relative limitations of this type of intervention.

**Method:**

This review summarizes the current evidence pooled from PubMed (Medline), EMBASE, EBSCOhost, http://clinicaltrials.gov, Scopus (Elsevier), Cochrane Central Register of Controlled Trials (CENTRAL), and Web of Science (Science Citation Index Expanded) databases for the use of stem cell therapies in stroke patients without combinations with other treatment modalities. The National Institutes of Health Stroke, modified Rankin Scales, and Barthel Index scores after external stem cell administration have been evaluated on the 3rd, 6th, and 12th months after treatment. The random effect analysis was performed using the Review Manager 5.4.1. The characteristics of stem cell sources and their adverse effects have been discussed as well.

**Findings:**

Although reasonably safe, the effectiveness evidence fluctuated to a large extent due to the heterogeneity of the clinical trials and the absence of a systematic approach. The stem cell sources and the administration window were not strongly associated with clinical outcomes.

**Conclusion:**

Further studies should be conducted to understand the deep discrepancy between preclinical and clinical trials and to execute phase 3 clinical trials with robust control of study characteristics and outcomes.

**Supplementary Information:**

The online version contains supplementary material available at 10.1007/s10072-023-07032-z.

## Introduction

Stroke is one of the leading causes of adult death and disability [1]. Despite advances in acute care, there is still no FDA-approved treatment option targeting neuroprotection following acute ischemic stroke, and the only acceptable therapy to promote early reperfusion remains the administration of tissue plasminogen activator and/or mechanical thrombectomy. These, given the short therapeutic window, are used only in the minority of cases and are not a treatment option for hemorrhagic stroke [2]. Even if the treatment protocols are constantly updated given the rapid advancement of endovascular techniques, certain procedures have provided clinical benefit within 6 h after symptoms onset [3]. The brain’s internal capacity to recover after stroke remains limited, although various mechanisms exist. Several trials to enhance the capacity of the brain to combat ischemic stroke were conducted [4]. These include the activation of the brain’s intrinsic defense mechanisms and external administration of stem and progenitor cells. From this perspective, stem cell therapy is an emerging therapeutic approach for stroke treatment. There is now a significant body of evidence coming from the preclinical data and clinical trials that stem cell administration has the potential to modulate multiple pathways arising from stroke. These include microenvironmental changes, reduction of the inflammatory response, and possible replacement of the injured tissue [5]․ However, the fundamental question remains, whether this therapy is safe and to what extent it can contribute to the recovery after stroke. Several systematic reviews were performed to address the effectiveness and usability of stem cell therapies for ischemic stroke. A. M. Abdullahi and colleagues analyzed the data of trials in the case of ischemic stroke, thus excluding hemorrhagic stroke trials, and the analysis included bone marrow-derived stem cell sources [6]. On another systematic approach, the authors broadened the stem cell sources, without explicitly analyzing the data for a specific time period [7]. In our review, we included the stem cell sources regardless of their source and marked the effects at 3-, 6-, and 12-month post-administration both in studies with and without comparator arms. The safety, feasibility, and effectiveness of external stem cell administration were evaluated.

## Methods

### Search strategy

Two independent reviewers performed the search using preliminarily defined keywords as shown on Table 1. The specific search strategies were created in consultation with a health sciences librarian with expertise in systematic review searches. The keyword selection was based on previously conducted reviews, as well as relevant keyword searches through scientific databases. After the PubMed strategy was finalized, it was adapted to the syntax and subject headings of the other databases. The databases included PubMed (Medline), EMBASE, EBSCOhost, http://clinicaltrials.gov, Scopus (Elsevier), Cochrane Central Register of Controlled Trials (CENTRAL), and Web of Science (Science Citation Index Expanded). In the event of differing interpretations, the two investigators re-evaluated the source material independently in an attempt to reach a consensus. If a consensus still could not be reached, a third, independent investigator was brought in to review the material and provide a decisive judgment.

**Table 1:** The search flowchart for PubMed

Search strategy for PubMed					
Population	Intervention	Route	Source	Exclusion (subject type)	Exclusion (article type)
Stroke (MeSH) OR	Stem cell (MeSH) OR	Transplant* OR	Autologous* OR	Animals (MeSH) OR	Review (publication type)
“Cerebrovascular accident” OR CVA OR	Stem cell transplantation (MeSH) OR	Implant* OR	Allogenic*	“Animal*” OR	
“Cerebrovascular insult” OR	“Stem cell*” OR	Infusion* OR		Mice (MeSH) OR	
“Brain attack” OR	“Mesenchymal stem cell*” OR	Neurosurg* OR		Mouse OR Mice OR	
“Intracerebral hemorrhage” OR	“Bone marrow stem cell*” OR	Intraven*		Rats (MeSH) OR	
“Cerebral hemorrhage” OR	“Hematopoietic stem cell*”			“Rat*”	
“Cerebral hematoma” OR					
“Brain hemorrhage” OR					
“Intracranial hemorrhage”					

(* serves as a wildcard for various term endings)

### Inclusion/exclusion criteria

The review evaluated the clinical trials, where patients, who had any kind of stroke, received stem cell therapy in the acute or chronic poststroke period, with any administration route, without combination with other therapeutic approaches. The safety measure included adverse effects (Table 2). The effectiveness measures were the National Institutes of Health Stroke Scale (NIHSS), modified Rankin Scale (mRS), and Barthel Index (BI) scores in baseline and at 3rd, 6th, and 12th months after treatment. The inclusion and exclusion criteria are shown in Table 2. The subgroups of the data were studies with and without a comparator arm.

**Table 2:** The inclusion/exclusion criteria

Selection criteria	
Inclusion criteria	Exclusion criteria
Original articles, trials, small-size studies	Reviews, narratives
Hemorrhagic and ischemic types of stroke	
Administration period: acute, subacute, or chronic phases of the stroke	
Only stem cell administration any type, by any route	Combination of stem cell administration with other therapies
Subjects: humans	Subjects: animals, cell culture
Tracking of adverse effects	
Adverse effects: definitely or probably related Adverse effects if seen regardless of the association with the administration: Seizures Hemorrhages Recurrent stroke Hemorrhagic transformation Life-threatening events	Adverse effects: possibly related or unrelated

### Meta-analysis workflow

The data were analyzed by the Review Manager 5.4.1 [8]. For the studies without a comparator arm, the data were used to calculate the change from baseline at 3, 6, and 12 months after treatment. The mean and the 95% confidence intervals were used to calculate the difference scores. The treatment effects for the controlled studies were explored at 3, 6, and 12 months. The baseline data for each arm was inspected to ensure that the groups did not significantly differ in NIHSS, BI, and mRS scores. To account for the potential heterogeneity across the studies, the random effects model has been used. Pooled estimates were presented as the mean difference (MD) with 95% confidence intervals. The respective forest plots were generated. Heterogeneity was summarized using the I-squared statistic in case of data was available from an adequate number of studies.

## Findings

### Study selection

**Fig. 1:**
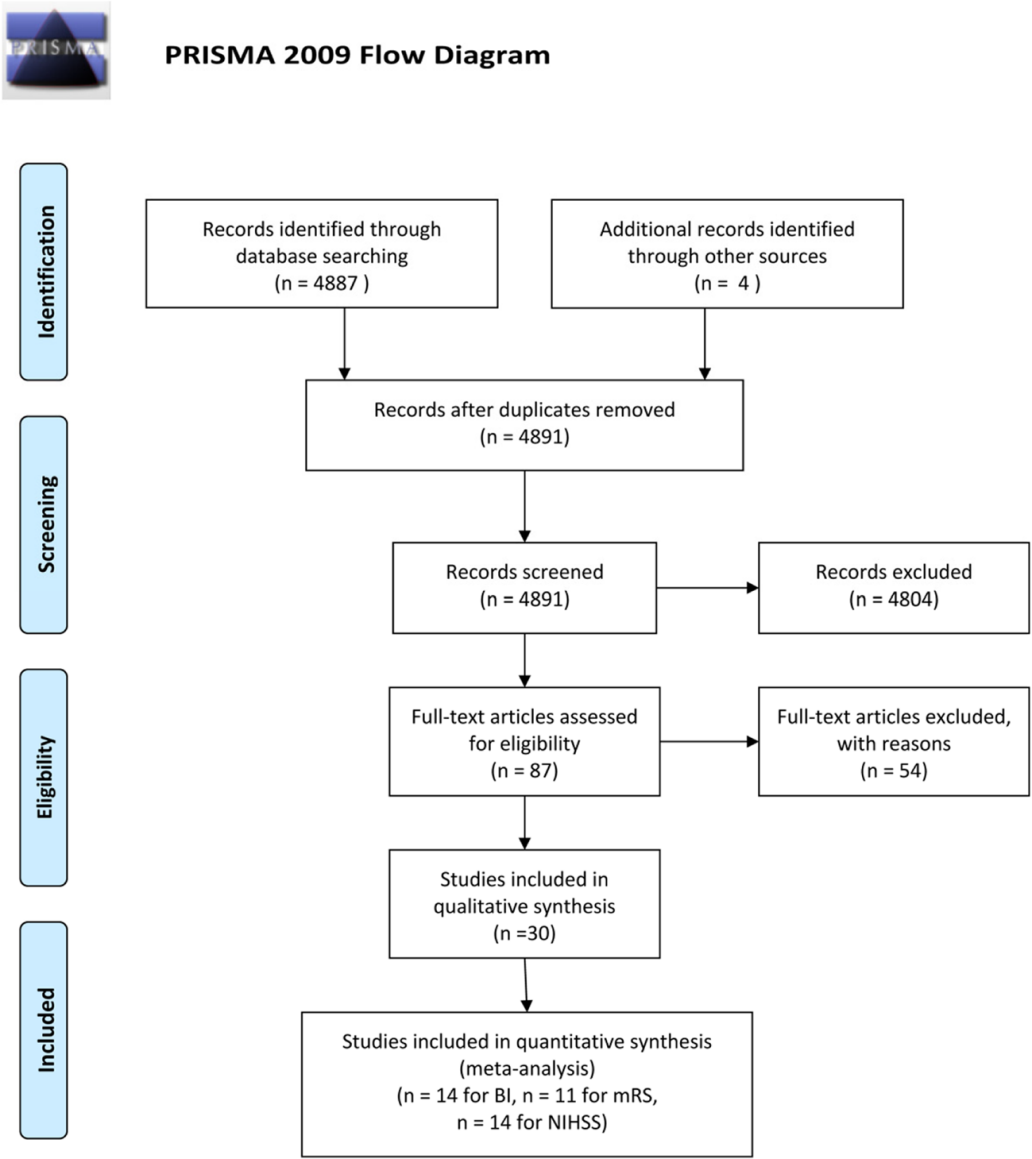
The PRISMA workflow. 14 studies for NIHSS, 14 studies for BI, and 11 studies for mRS were used for quantitative synthesis

Preferred Reporting Items for Systematic Reviews and Meta-Analyses (PRISMA) guidelines were used to select the articles [9]. 30 articles, that fulfilled the inclusion criteria, were included for qualitative analysis (Fig. 1) [4, 10–38]. 14 studies for NIHSS, 14 studies for BI, and 11 studies for mRS fulfilled the criteria for quantitative synthesis (Fig. 1).

### Patient baseline characteristics

All patients, with the exception of those in the study by [39], received acute treatment for stroke based on clinical characteristics and imaging findings. The majority of trials involved patients from both genders with an age range from 3 and 85 years. The mean age of patients (mean ± SD if available), age range, and number or patients in each included study are summarized in supplementary material 1. Among the studies included in our analysis, only the cohort from Tsang KS. et al. (2017) comprised patients with hemorrhagic stroke. Meanwhile, the study from Jiang Y. et al. (2013) had one patient with a hemorrhagic stroke, Chen L. et al. (2013) had four such patients, Suarez-Monteagudo C et al. (2009) had three, and Rabinovich SS et al. (2005) also had three patients. All other patients had ischemic stroke. The most relevant exclusion criteria for the treatment were lacunar syndrome, any severe condition likely to interfere the treatment, malignant disorders, pregnancy, HIV positivity, prior immunosuppression, participation in another investigational drug or device study, contraindication to imaging studies, severe hemorrhagic transformation of the ischemic lesion, and unwillingness to participate (supplementary material 1).

### Analysis of the type of stem cells used in studies and study characteristics

The majority of studies (10) have used bone marrow-derived mononuclear cells (BM-MSCs) for the studies [10–13, 17, 19, 25, 32–34, 39], where all of the cell sources were autologous. Eight studies utilized bone marrow-derived mesenchymal stem cells (BM-MesSCs) [13, 15, 16, 18, 26, 28, 29, 38]; six of the cell sources were autologous, while two were allogenic [18, 38]. Remaining trials used CTX-DP immortalized human neural stem cell line [37], multipotent adult stem cells [35], umbilical cord blood MesSCs [22, 23, 31], enriched population of aldehyde dehydrogenase-bright stem cells (ALD-401) [4], neural stem/progenitor cells (NSPCs) [22] and bone marrow-derived mesenchymal stem cells (BM-MesSCs) [30], CD34+ hematopoietic stem/progenitor cells [24, 27], immature nerve cells and hemopoietic hepatic cells [21], LBS neurons [20], endothelial progenitor cells [17], MultiStem (HLM051) [14], olfactory ensheathing cells (OEC), and Schwann cells (SC) [22] (Table 3). Further details, such as administration route and administration window, are provided in supplementary material 2.

**Table 3:** The stem cell types used in clinical trials. Computations for each cell source are conducted independently, including the studies utilizing multiple stem cell sources

Cell source	Number of studies	Abbreviations
BM-MSCs	10	NSPCs—neural stem/progenitor cells BM-MesSCs—bone marrow-derived mesenchymal stem cells BM-MSCs—bone marrow-derived mononuclear stem cells IN—immature nerve cells HHCs—hemopoietic hepatic cells CTX-DP—immortalized human neural stem cell line M-APCs—multipotent adult progenitor cells ALD-401—aldehyde dehydrogenase-bright stem cells EPCs—endothelial progenitor cells OECs—olfactory ensheathing cells SCs—Schwann cells PBSCs—peripheral blood stem cells
BM-MesSCs	8	
M-APCs	1	
CTX-DP	1	
Umbilical cord MesSCs	3	
CD34+ PBSCs	2	
MultiStem (HLM051)	1	
EPCs	1	
LBS neurons	1	
IN and HHCs	1	
OECs, SCs, and umbilical cord MesSCs	1	
NSPCs and BM-MesSCs	1	
ALD-401	1	

The most common route of cell administration was intravenous (15), and 6 studies have used intracerebral route for cell administration. In 5 studies, the cells were administered using intra-arterial route, 1 subarachnoid (spinal) and 1 intrathecal. The remaining studies used combinatorial approach (2).

The number of patients included in a specific type of trial and the distribution of the trials based on their design are shown in Table 4.

**Table 4:** The distribution of patients based on clinical study design

Type of the trial	Number of screened patients	Number of included patients	Number of patients receiving intervention
Randomized, double-blind, placebo-controlled trial	493	427	214
Randomized, open-label, blinded–end point study	229	20	10
Non-randomized, open label study	169	446	205
Non-randomized, open label, blinded–end point study	379	18	18
Open-label trial with observer-blinded evaluation of patients	12	12	12
Randomized, open-label, observer-blinded clinical trial	85	52	16
Randomized, parallel group trial with blinded outcome assessment	433	120	60
Randomized, single blind, placebo- controlled clinical trial	183	199	99
Total	1983	1294	634

### Effectiveness

#### National Institutes of Health Stroke Scale (NIHSS) score

**Fig. 2:**
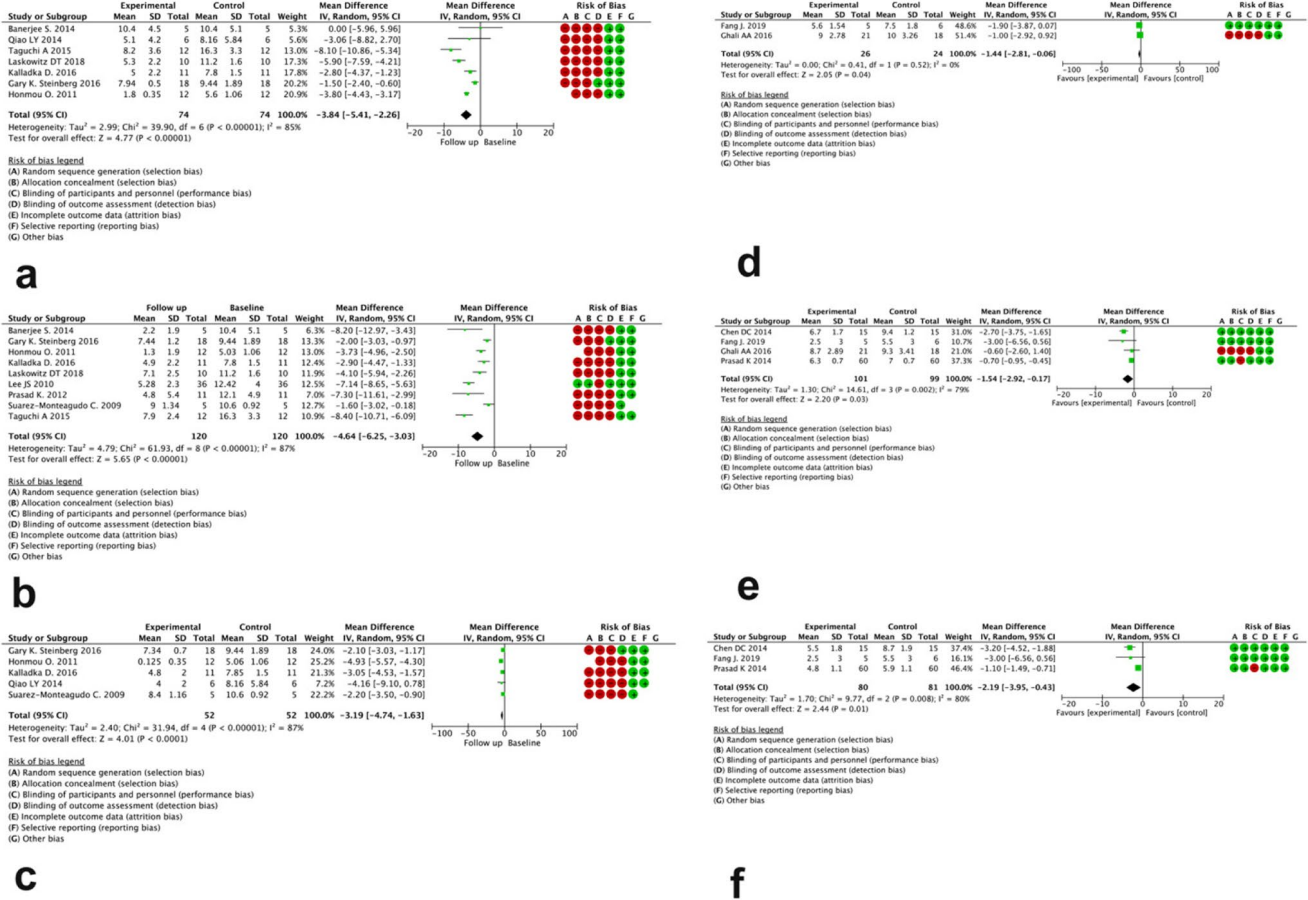
Forest plot of post-treatment NIHSS score change after 3, 6, and 12 months in studies without (**a**, **b**, **c**) and with comparator arm (**d**, **e**, **f**) respectively

We have evaluated available data from seven single-arm studies that reported the impact of stem cell therapies at 3-, 6-, and/or 12-month post-treatment. The NIHSS scores decreased in a modest manner after 3 (MD = −3.84 (95% CI −5.41 to −2.26)) and 6 (MD = −4.64 (95% CI −6.25 to −3.03)) months (Fig. 2a, b). In four studies, the change of NIHSS score was also available after 12 months post-treatment (MD = −3.19 (95% CI −4.74 to −1.63)) (Fig. 2c). The I2 scores were 85, 87, and 87%, respectively, indicating significant heterogeneity across the studies. NIHSS decrement in the studies having comparator arm was as follows: MD = −1.44 (95% CI −2.81 to −0.06), MD = −1.54 (95% CI −2.92 to −0.17), and MD = −2.19 (95% CI −3.95 to −0.43) after 3, 6, and 12 months, respectively (Fig. 2d, e, f). The studies had significant heterogeneity.

#### Barthel Index (BI) score

**Fig. 3:**
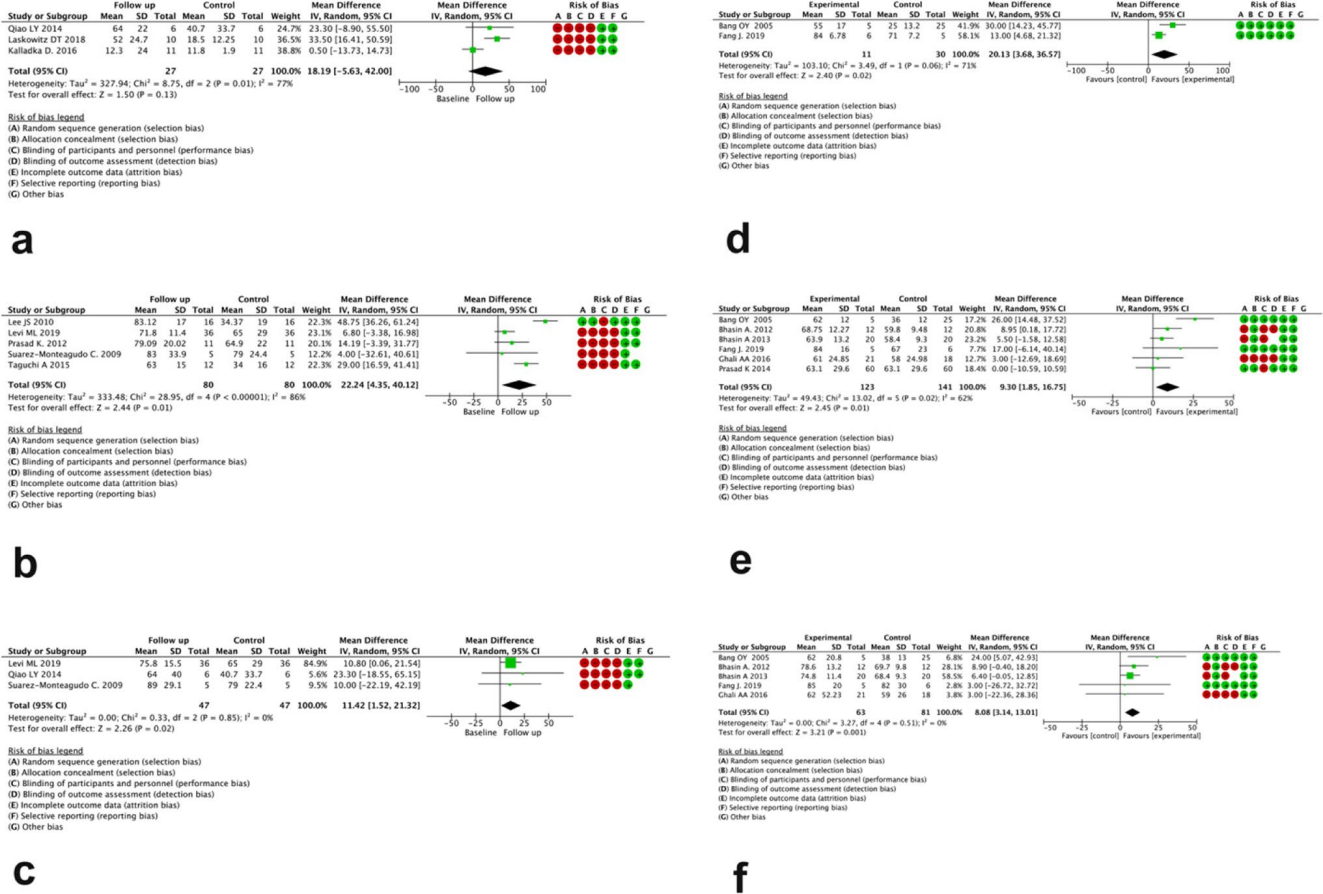
Forest plot of post-treatment BI score change after 3, 6, and 12 months in studies without (**a**, **b**, **c**) and with comparator arm (**d**, **e**, **f**), respectively

The BI change in follow-up studies without a comparator arm was as follows: MD = 18.19 (95% CI -5.63 to 42.00), MD = 22.24 (95% CI 4.35 to 40.12), and MD = 11.42 (95% CI 1.52 to 21.32) after 3, 6, and 12 months, respectively (Fig. 3a, b, c). studies (I score > = 77%). BI change in the studies having comparator arm was as follows: MD = 20.13 (95% CI 3.68 to 36.57), MD = 9.30 (95% CI 1.85 to 16.75), and MD = 8.08 (95% CI 3.14 to 13.01) after 3, 6, and 12 months, respectively (Fig. 3d, e, f). The studies had significant heterogeneity as in the previous case, however being moderate (I score = 62%) at 6-month evaluation.

#### Modified Rankin Scale (mRS) score

**Fig. 4:**
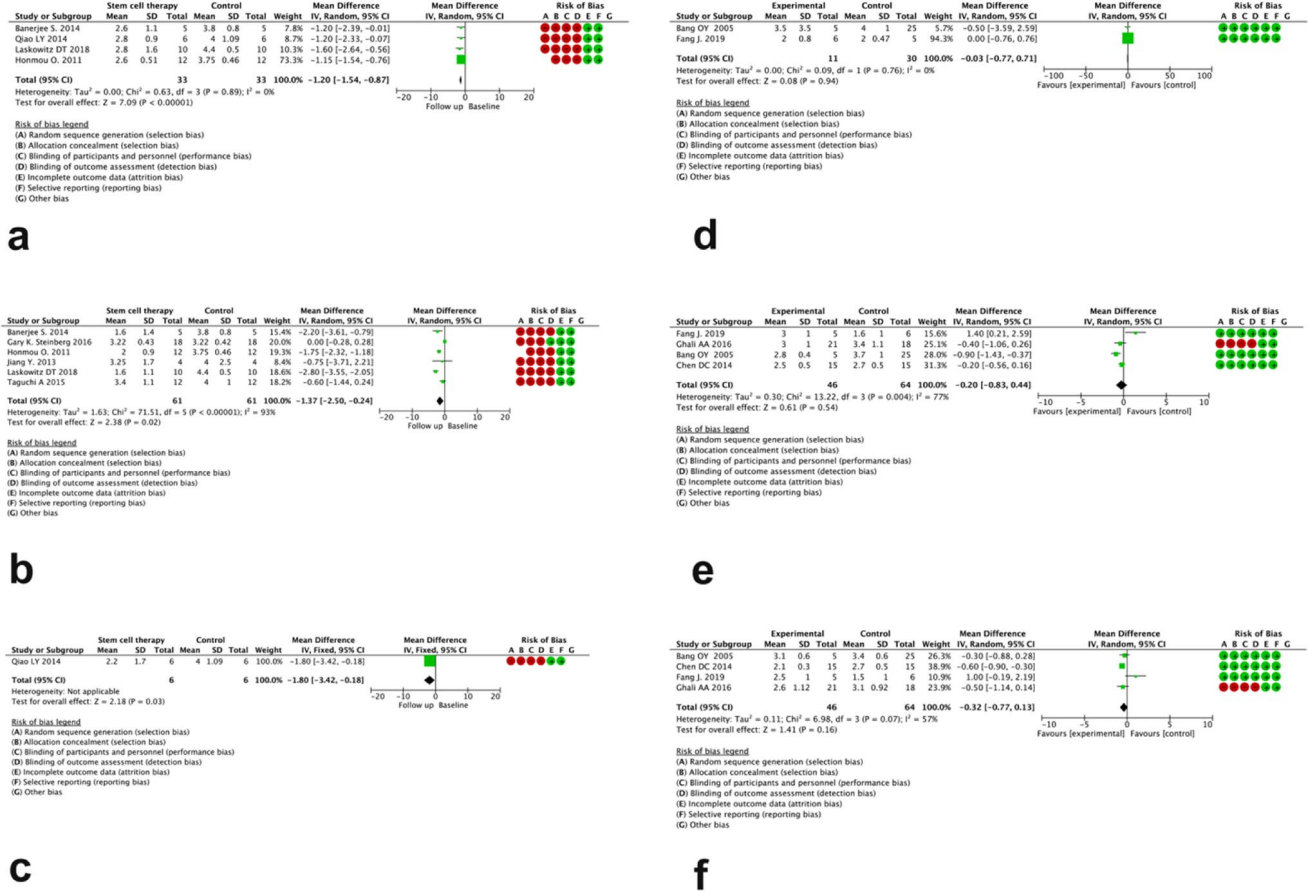
Forest plot of post-treatment mRS score change after 3, 6, and 12 months in studies without (**a**, **b**, **c**) and with comparator arm (**d**, **e**, **f**), respectively

The mRS change in follow-up studies without a comparator arm was as follows: MD = −1.20 (95% CI −1.54 to −0.87), MD = −1.37 (95% CI −2.50 to −0.24), and MD = −1.80 (95% CI −3.42 to −0.18) after 3, 6, and 12 months, respectively (Fig. 4a, b, c). There was significant heterogeneity across studies (I score > = 92%). As for the studies with the comparator arm, the mRS change was as follows: MD = −0.03 (95% CI −0.77 to 0.71), MD = −0.20 (95% CI −0.83 to 0.44), and MD = −0.32 (95% CI −0.77 to 0.13), and I2 was 77 % and 57 % after 3, 6, and 12 months, respectively (Fig. 4d, e, f).

### Risk of bias assessment

**Fig. 5:**
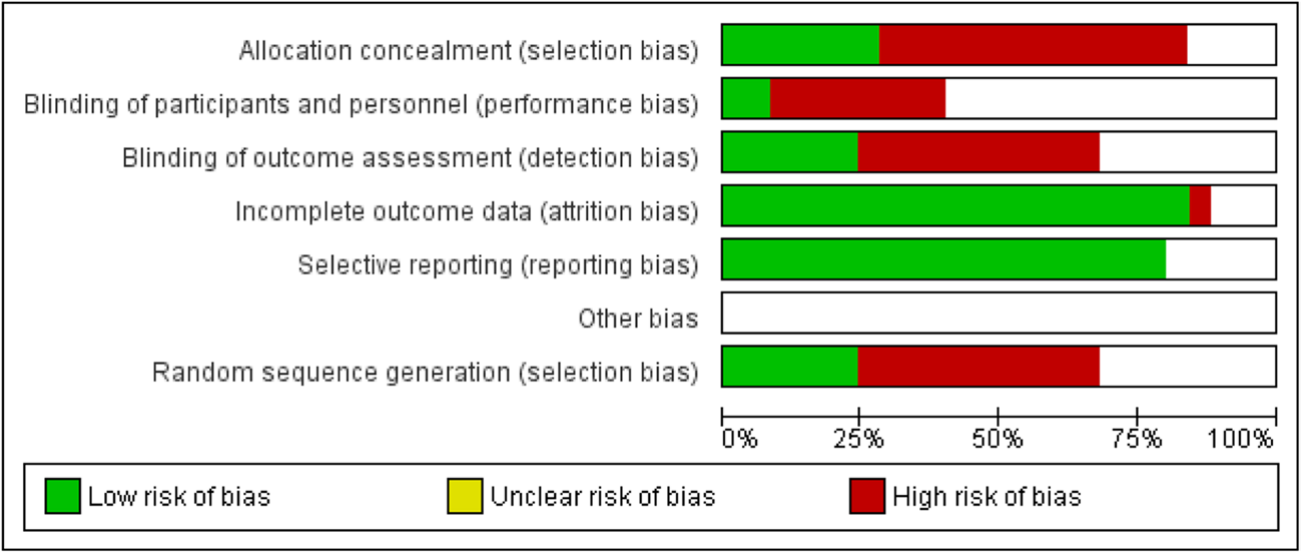
Risk of bias summary. The cumulative risk of bias pooled from included studies

The allocation concealment was adequate in 7 studies, and random sequence generation was reported in 6 studies. Only two studies have used blinding of the participants and study personnel. There was only one case when the reviewers doubted the completeness of the outcomes in comparison with per-protocol data. The bias of other sources was hard to evaluate. The cumulative risk of bias table is shown on Fig. 5.

### Adverse events

Seven cases of subdural and one extradural hematoma were observed which were assumed as related to the stem cell administration [4, 18, 37, 38]. The course was asymptomatic and without any neurological deterioration. One patient developed small infarct which was clinically silent [11]. In eight cases, the personnel observed a convulsion state among the patients, which was considered as non-related [4, 20, 38]. Five in control and 3 in experimental had seizures after stroke; 3 and 4 patients developed vascular recurrent stroke in control and experimental groups, respectively [28]. Two cases of recurrent stroke were observed [20, 33]. In the later, the relationship was considered as unclear. One case of serious deterioration in treatment group has been reported [34]. No other serious treatment-emergent adverse events were reported, or life-threatening adverse events and death were assessed to be not significantly different between the studies having comparator arm. No intracranial tumor formation during the study period was reported. Other events during the follow-up period were reported to be self-limiting and without any complications (supplementary material 2).

## Discussion

The review evaluates up-to-date evidence of the usage of stem cell therapies for the improvement of clinical outcomes after stroke. Over the last 10 years, there is a strong interest in the potential of stem cells as a therapeutic modality after stroke. Although increasing in numbers, the studies lack a standardized approach, which makes it difficult to draw critical assumptions for the effectiveness of these therapies. Considering that the studies included in the analysis had substantial heterogeneity and variability in results, we have compared them based on different characteristics.

One of the most relevant parameters affecting the study outcome was the study design. As the non-comparator arm studies inherently lack a control arm, there is no baseline for comparison to ascertain whether the improvements were the consequence of the intervention or the result of standard care and brain repair mechanisms. The inclusion of a comparator arm, in contrast to a historical control or control to baseline, reduced the cases with significant improvement. As shown in Figs. 2b, 3b, and 4b, the improvement in NIHSS, BI, and mRS after 6 months in the studies without a comparator arm was observed in 11 out of 14 cases; however, the improvement of those parameters after 6 months was observed in only 5 out of 13 measurements in studies with a comparator arm (Figs. 2e, 3e, and 4e). Another observable point is that studies without a comparator arm show less improvement at 12 months when compared to 6 months (Figs. 2c, 3c, and 4c). This could be due to the fact of brain recovery at 12 months independent of the therapy. The evaluation at 3 months was not significantly different from those at 6 and 12 months, with the exception of the BI change, which was notably higher in studies featuring a comparator arm.

Interestingly, NIHSS reduction resulted in more significant improvement when compared to the cases when BI was implemented (Figs. 2 and 3). This can be explained by the different variables those tests measure. Whereas NIHSS checks neurological parameters like visual fields, facial palsy, and arms and legs drift, the BI focuses on functional parameters like independence in bathing, grooming, dressing, and toilet use. Consequently, NIHSS may be a more sensitive test in detecting improvements; however, those improvements may not be enough to significantly affect disability and functional recovery.

The studies with a comparator arm mainly used an intravenous injection of autologous stem cells with various outcomes. Nevertheless, one study with an intracerebral injection of autologous stem cells demonstrated improved outcomes both after 6 and 12 months [24]. On the other hand, the study with intra-arterial injection did not result in an improved outcome in any of the parameters.

We have analyzed studies with allogenic stem cells but none of them included a comparator arm. Therefore, we expect the results of upcoming clinical trials to see the effect of allogenic stem cells on restoration after stroke.

We also questioned why studies with a comparator arm using intravenous injection with autologous stem cells resulted in different outcomes (Fang et al. 2014-negative, Bang et al. 2005-positive, Prasad et al. 2014, Bhasin et al 2012, 2013-mixed). The difference may underlie in the study design, patient selection, stem cell preparation, and other variables.

The adverse effects reported in trials were self-limited or resolved after appropriate treatment. There was no alarming signal in relation to tumorigenicity and recurrent stroke. The reported adverse effects were mainly associated with the administration procedure, and the course was free of complications. The results, however, are self-limited and exhibit variability with respect to the duration of follow-up periods and the strategies employed for evaluation.

## Future perspectives

Stem cell therapies act via multiple mechanisms, including the release of growth factors, anti-inflammatory effects, and possibly exosomes [18]. Although the regeneration mechanisms of the brain toward injury are still active long after stroke onset, they are insufficient to fully recover from damage. The synaptic plasticity changes, reorganization of existing neural circuits, and cell genesis, however, may be benefited from external stem cell administration. In our review, there is a non-significant trend toward improvement of some functional parameters included in the meta-analysis. However, the small extent of the improvement and the alarming level of heterogeneity across the studies arises a significant body of questions needed to be addressed in future clinical trials. The studies having comparator arm lacked a randomization approach per se, and the blinding procedure of the personnel and the study participants was missing in the majority of cases. In our opinion, such higher heterogeneity can be also due to a wide range of stem cell sources, administration route and window (supplementary materials 1 and 2). The time interval between the onset of stroke and cell therapy may be important in terms of the efficacy. When choosing the appropriate cell type and administration window, the majority of studies referred to previously reported findings on rodent models, although the extrapolation of these findings may not be always straightforward. Additionally, the optimal dose of stem cells is largely unknown. In this context, further studies should be conducted to understand the deep discrepancy between preclinical and clinical trials and execute phase 3 clinical trials with robust control of study characteristics and outcomes.

The review limitations include a relatively high percentage of small-size studies among others, a high extent of heterogeneity of stem cell usage, window and administration route, lack of opportunity to conduct subgroup analysis, and inherent obstacles coming from the non-availability of the additional information on request.

### Supplementary information

ESM 1 (DOCX 46.9 kb)

ESM 2 (DOCX 38.5 kb)

## Data Availability

The data can be available upon reasonable request.
