# Supplementary material for: A review and meta-analysis of stem cell therapies in stroke patients: effectiveness and safety evaluation
Source: Neurol Sci. 2023 Sep 21;45(1):65–74. doi: 10.1007/s10072-023-07032-z (PMC10761518; doi:10.1007/s10072-023-07032-z)
Supplement: Supplementary file 1 — (DOCX 46.9 kb) [file 10072_2023_7032_MOESM1_ESM.docx]

| **First author** | **Title** | **Study type** | **Patient characteristics (***mean± SD years* for age if available**)** | **Stroke type** | **Most relevant exclusion criteria** |
| --- | --- | --- | --- | --- | --- |
| Savitz Si et al ., 2019 | A Phase 2 Randomized, Sham-Controlled Trial of Internal Carotid Artery Infusion of Autologous Bone Marrow-Derived ALD-401 Cells in Patients With Recent Stable Ischemic Stroke (RECOVER-Stroke) | randomized, double-blind, placebo-controlled, multicenter trial | - 59.3 ±10.03 in experimental and 62.9 ±10.81 in control arms - Age range*:* 30-83 years - Men and women - 29 patients in the intervention and 16 in control groups | ischemic stroke | - unable to undergo the bone marrow harvest - unable to undergo angiography - clinically significant intracranial hemorrhage - subjects with previous disability with mRS ≥ 1 before stroke |
| Vahidy FS et al., 2019 | Intravenous Bone Marrow Mononuclear Cells for Acute Ischemic Stroke: Safety, Feasibility, and Effect Size from a Phase I Clinical Trial | non-randomized, open label study | - 60.7 ±13.3 in experimental and 63.7 ±12.5 in control arms - Men and women - 25 patients in the intervention and 185 in control groups | ischemic stroke | - subjects with previous disability with mRS ≥ 1 before stroke - if patient is > 80 years old - ischemic stroke in the past 3 months - myocardial infarction in the past 3 months - mechanical heart valve - primary hemorrhagic or traumatic liaison of the brain within the last 3 months - seizure disorder - pregnancy - chronic kidney and hepatic diseases - active malignancy - prior immunosuppression, including chemotherapy - uncorrected coagulopathy at the time of consent - any hemodynamic instability at the time of consent - unable to undergo MRI or CT scan |
| Levy ML et al., 2019 | Phase I/II Study of Safety and Preliminary Efficacy of Intravenous Allogeneic Mesenchymal Stem Cells in Chronic Stroke | non-randomized, open label study | - 61.1 ± 10.8 - Men and women - Age range: 39-84 years - 36 patients | ischemic stroke | - history of uncontrolled seizure disorder - history of cancer within the past 5 years - history of cerebral neoplasm - positive for hepatitis B, C, or HIV - myocardial infarction within 6 months of study entry - presence of any other clinically significant medical or psychiatric condition, or laboratory abnormality - findings on baseline computed tomography suggestive of subarachnoid or intracerebral hemorrhage within past 12 months - participation in another investigational drug or device study in the 3 months before treatment - pregnant or lactating - allergy to bovine or porcine products |
| Fang J. et al., 2019 | Autologous Endothelial Progenitor Cells Transplantation for Acute Ischemic Stroke: A 4-Year Follow-Up Study | randomized, single blind, placebo- controlled clinical trial | - 50.80 ± 10.14 in experimental and 52.8 ± 14.95 in control arms - Age range: 18-80 years - Men and women - 5 patients in the intervention and 6 in control groups | ischemic stroke | - lacunar syndrome - diagnosis other than ischemic stroke - hematological causes of stroke - severe respiratory, hepatic, or renal disorders - presence of severe febrile illness or viral diseases - malignant diseases - presence of autoimmune diseases - positive response of penicillin - skin pregnancy |
| Bhatia V. et al., 2018 | Randomized Assessment of the Safety and Efficacy of Intra-Arterial Infusion of Autologous Stem Cells in Subacute Ischemic Stroke | randomized, open-label, blinded–end point study | - 66± 7.3 in control and 57± 12.2 in experimental arms - Age range: 20–80 years - Men and women - 10 patients in intervention and 10 in control groups | ischemic stroke | - cerebral hemorrhage on CT/MR imaging - imaging evidence of M1 MCA segment complete occlusion - hemodynamic instability, known defect of clotting - severe comorbidity precluding intra-arterial intervention - hepatic dysfunction, renal dysfunction - pregnancy - patients likely to be unavailable for follow-up, patients with evidence of chronic illness or advanced cancer - refusal to give informed consent |
| Laskowitz DT et al., 2018 | Allogeneic Umbilical Cord Blood Infusion for Adults with Ischemic Stroke: Clinical Outcomes from a Phase I Safety Study | non-randomized, open label study | - Median age: 65.5 - Age range: 45–79 years - Only men - 10 patients | ischemic stroke | - immunocompromised patients - patients with a medical history of neurological or orthopedic pathology - pre-existing cognitive deficit - evidence of midline shift, edema, or mass effect   and serious psychiatric or neurological disease   - malignancy or autoimmune disease |
| Deng L. et al., 2018 | Intrathecal Injection of Allogenic Bone Marrow-Derived Mesenchymal Stromal Cells in Treatment of Patients with Severe Ischemic Stroke: Study Protocol for a Randomized Controlled Observer-Blinded Trial | randomized, single blind, placebo- controlled clinical trial | - 59 patients in each group | ischemic stroke | - cerebral infarction due to special causes - recurrent stroke within 3 months combined with hemorrhagic transformation, cerebral hemorrhage, or subarachnoid hemorrhage - severe disorders of consciousness (lethargy or coma) - cerebral hernia - status epilepticus - severe medical condition: multiple organ failure, unstable vital signs - autoimmune diseases - presence of other neurological diseases, dementia, or mental illness prior to the current stroke that is likely to confound clinical evaluation and understanding of the informed consent - cancer - pregnant or lactating women - allergy to local anesthetic - current participation in another clinical trial or participation in another clinical trial within 30 days |
| Osanai T. et al., 2017 | Treatment evaluation of acute stroke for using in regenerative cell elements (TREASURE) trial: Rationale and design | randomized, double-blind, placebo-controlled trial | - Over 20 years old - 110 patients in each arm (preliminary) | ischemic stroke | - lacunar infarct - reduced level of consciousness - occurrence of a hemorrhagic transformation - ipsilateral focal neurological deficits from prior lesions in the brain that would complicate evaluation - experienced seizures since the onset of ischemic stroke - history of a neurological event such as stroke - patients who both received tPA treatment and underwent mechanical reperfusion - uncontrolled hypertension - severe organ dysfunctions - known human immunodeficiency virus infection - Alzheimer’s disease or other dementias - history of malignant tumor - contraindication for MRI - prior participation in another clinical trial involving investigational pharmacological agents or devices within 30 days prior to providing consent |
| Tsang KS. et al., 2017 | Phase I/II randomized controlled trial of autologous bone marrow-derived mesenchymal stem cell therapy for chronic stroke | randomized, double-blind, placebo-controlled trial | - Mean age: 52 - Age range: 41-59 years - 5 patients in intervention and 4 in control groups | intracerebral hemorrhage | - lacunar syndrome - malignant diseases - severe co-morbidity, hepatic/renal dysfunction - unwillingness to participate |
| Hess David C. et al., 2017 | Safety and efficacy of multipotent adult progenitor cells in acute ischaemic stroke (MASTERS): a randomised, double-blind, placebo-controlled, phase 2 trial | randomized, double-blind, placebo-controlled trial | - 61.8± 11.4 in experimental and 62.6 ± 11.4 in control arms - Age range: 17-83 years - Men and women - 63 patients in intervention and 60 in control groups | ischemic stroke | - brainstem or lacunar infarct - severe congestive heart failure - chronic obstructive pulmonary disease - renal or hepatic failure - inability to undergo an MRI scan history of splenectomy |
| Shichinohe H. et al., 2017 | Research on advanced intervention using novel bone marrow stem cell (RAINBOW): a study protocol for a phase I, open-label, uncontrolled, dose-response trial of autologous bone marrow stromal cell transplantation in patients with acute ischemic stroke | non-randomized, open label study | - Age range: 20-79 years - Men and women - 6 patients | ischemic stroke | - severe hemorrhagic transformation of ischemic stroke - deep coma - severe anemia (hemoglobin < 10.0 g/dL), thrombocytopenia (platelet count < 100,000/mm3), or severe heart disease - uncontrolled hypertension, history of any malignancy, or carriers of certain infectious diseases (Syphilis, HBV, HCV, HIV-1/2, HTLV-1, or parvovirus B19) - pregnant individuals - MRI contraindications |
| Steinberg Gary K. et al., 2016 | Clinical Outcomes of Transplanted Modified Bone Marrow–Derived Mesenchymal Stem Cells in Stroke | non-randomized, open label, blinded–end point study | - 61.3± 10.29 - Age range: 33-75 years - Men and women - 18 patients (3 cohorts-different doses) | ischemic stroke | - history of >1 symptomatic stroke   • presence or history of any other major neurological  disease  • cerebral infarct size >100 cm3 measured by MRI scan  • myocardial infarction in the past 6 months  • history of central nervous system malignancy  • history of seizures or current use of antiepileptic  medication  • uncontrolled systemic illness   - significant laboratory test abnormalities - pregnant individuals - MRI contraindications - acute intracranial hemorrhage |
| Kalladka D. et al., 2016 | Human neural stem cells in patients with chronic ischaemic stroke (PISCES): a phase 1, first-in-man study | non-randomized, open label study | - Median (IQR) 68 (61–75) - Age range: 60 -82 years - Men - 11 patients in 4 cohorts | ischemic stroke | - any unstable disorder   with expected survival shorter than 12 months   - major surgery in the previous 30 days - history of epilepsy or a blood coagulation disorder - had undergone allogeneic stem cell   tissue, organ, or bone-marrow transplantation   - contraindication to MRI - patients taking tamoxifen |
| Ghali AA et al., 2016 | Intra-arterial Infusion of Autologous Bone Marrow Mononuclear Stem Cells in Subacute Ischemic Stroke Patients. | non-randomized, open label study | - Age range: 46 - 66 years - Men and women - 21 patients in the experimental and 18 patients in the control group | ischemic stroke | - cardio-embolic stroke - severe carotid stenosis (>70%) - primary hematological disease, neurodegenerative disorder - previous stroke with modified Rankin Scale (mRS) >2 - auto-immune disorders - liver failure, chronic renal failure - lacunar stroke |
| Bhasin A. et al., 2016 | Paracrine Mechanisms of Intravenous Bone Marrow-Derived Mononuclear Stem Cells in Chronic Ischemic Stroke. | randomized, single blind, placebo- controlled clinical trial | - 48.6 ± 7.1 - Men and women - 20 age matched patients in each group | ischemic stroke | - hematological disorders, autoimmune disorders, immunocompromised subjects’ chronic liver and renal failure, progressive neurological worsening - neoplasia - contraindication to MRI - pregnancy |
| Taguchi A. et al.,2015 | Intravenous Autologous Bone Marrow Mononuclear Cell Transplantation for Stroke: Phase1/2a Clinical Trial in a Homogeneous Group of Stroke Patients | non-randomized, open label study | - 67.4 ± 5.4 - Age range: 20-75 years - Predominantly males - 12 (2 cohorts) patients | ischemic stroke | - expected brain surgery; acute myocardial infarction - coagulation disorders - uncontrolled proliferative diabetic retinopathy - infective endocarditis - positivity for hepatitis B virus, hepatitis C |
| Prasad K. et al., 2014 | Intravenous autologous bone marrow mononuclear stem cell therapy for ischemic stroke: a multicentric, randomized trial | randomized, parallel group trial with blinded outcome assessment | - 50.7+11.6 in treatment and 52.5+12.1in control arms - Men and women - 60 patients in both arms | ischemic stroke | - lacunar syndrome - intubation - posterior circulation stroke - co-morbidity - pre-stroke disability - inaccessibility for follow up - allergy to local anaesthetic - unwillingness to provide written informed consent - symptoms suggestive of acute cardiac, hepatic or renal disease - pregnancy - HIV positivity, or participation in any other trial |
| Qiao LY et al., 2014 | A two-year follow-up study of cotransplantation with neural stem/progenitor cells and mesenchymal stromal cells in ischemic stroke patients | non-randomized, open label study | - 56,1+ 29.9 - Age range: 3- 85 years - Men and women - 6 patients | ischemic stroke | - comorbidity that was likely to limit survival to less than 3 years - a hemisphere damaged by a previous stroke - pregnancy - HIV positivity |
| Banerjee S. et al., 2014 | Intra-Arterial Immunoselected CD34+ Stem Cells for Acute Ischemic Stroke | non-randomized, open label study | - 58.2+12.8 - Age range: 30–80 years - Men and women - 5 patients | ischemic stroke | - known defect of clotting - hematological causes of stroke - severe comorbidity - hepatic dysfunction - female patients of childbearing potential or breast feeding - patient is likely to be unavailable for follow-up - patients with evidence of life-threatening infection (e.g., HIV) or life-threatening illness (e.g., advanced cancer) - patient already dependent in activities of daily living before the present acute stroke - patients who were included in any other clinical trial within the previous month |
| Chen DC et al., 2014 | Intracerebral implantation of autologous peripheral blood stem cells in stroke patients: a randomized phase II study | randomized, single blind, placebo- controlled clinical trial | - 50.1 ± 7.7 in experimental and 52.8 ± 9.0 in control arms - Age range: 35-75 years - Men and women - 15 patients in each group | ischemic stroke | - confounding disorders - pregnancy |
| Jiang Y. et al., 2013 | Feasibility of delivering mesenchymal stem cells via catheter to the proximal end of the lesion artery in patients with stroke in the territory of the middle cerebral artery | non-randomized, open label study | - Age range: 40 -59 years - Men - 4 patients | ischemic and one 1 case of hemorrhagic stroke | - uncontrolled infection, failure of one of the vital organs - intracranial neoplasms - acute myocardial infarction - pregnancy - allergy to fetal products or iodine |
| Chen L. et al., 2013 | Multiple cell transplantation based on an intraparenchymal approach for patients with chronic phase stroke | non-randomized, open label study | - 55.00 ± 14.38 - Age range: 42-87 years - Men and women - 10 patients | 6 ischemic and 4 hemorrhagic cases | - not available |
| Prasad K. et al., 2012 | Autologous intravenous bone marrow mononuclear cell therapy for patients with subacute ischaemic stroke: a pilot study | non-randomized, open label study | - 51.45± 11.77 - Age range: 30- 70 years - Men and women - 11 patients | ischemic stroke | - lacunar syndrome, intubation - likely to limit survival to less than three years - malignant diseases - hepatic or renal failure - pre-stroke disability leading to dependence on others for activities of daily living - inaccessibility for follow up - pregnancy or HIV positivity |
| Bhasin A. et al., 2012 | Autologous intravenous mononuclear stem cell therapy in chronic ischemic stroke | non-randomized, open label study | - 45.05± 12.1 in treatment and 45.45±9.7 in control arms - Age range: 18–65 years - Predominantly men - 20 patients in each group | ischemic stroke | - autoimmune disorders, immune-compromised states, hematological disorders - chronic liver and renal failure - progressive neurological worsening - neoplasia - contraindication to MRI - pregnancy |
| Honmou O. et al., 2011 | Intravenous administration of auto serum-expanded autologous mesenchymal stem cells in stroke | non-randomized, open label study | - 59.2± 8.2 - Age range: 20-75 years - Men and women - 12 patients | ischemic stroke | - severe hemorrhagic transformation of ischaemic lesion - severe consciousness disturbance - pregnant or possibly pregnant - severe medical complications such as kidney dysfunction, liver dysfunction - malignant diseases - severe ischaemic diseases - severe anemia - strokes that were infratentorial - severe comorbidies |
| Lee JS et al., 2010 | A long-term follow-up study of intravenous autologous mesenchymal stem cell transplantation in patients with ischemic stroke | randomized, open-label, observer-blinded clinical trial | - 64.9 ± 14.5 in experimental and 64.0 ± 11.6 in control groups - Age range: 30-75 years - Men and women - 16 in experimental and 36 in control groups | ischemic stroke | - lacunar infarction - presence of severe medical illness - presence of severe febrile illness - hepatic or renal dysfunction - positive response of penicillin skin test - unwillingness to participate |
| Bang OY et al., 2005 | Autologous mesenchymal stem cell transplantation in stroke patients | randomized, double-blind, placebo-controlled trial | - 59.3 ± 11.5 in control and 63.0 ±   7.5 in experimental arm - Age range: 30-75 years - Men and women - 5 experimental and 25 in control group | ischemic stroke | - lacunar syndrome, hematological causes of stroke, malignant diseases, severe comorbidity - hepatic or renal dysfunction - unwillingness to participate |
| Suarez-Monteagudo C et al., 2009 | Autologous bone marrow stem cell neurotransplantation in stroke patients. An open study | non-randomized, open label study | - 51.4±9.2 - Men and women - Age range: 40-70 years - 5 patients | 3 ischemic and 2 hemorrhagic stroke cases | - chronic diseases - severe cognitive - deterioration, psychiatric diseases patients with - more than one brain lesion |
| Rabinovich SS et al., 2005 | Cell therapy of brain stroke |  | - 35-56 years - Men and women - 10 patients | 7 ischemic and 3 hemorrhagic stroke cases | - not available |
| Kondziolka D. et al. 2000 | Transplantation of cultured human neuronal cells for patients with stroke | open-label trial with observer-blinded evaluation of patients | - Age range: 44 -75 years - Men and women - 12 patients | ischemic stroke | - not available |
