# Supplementary material for: A review and meta-analysis of stem cell therapies in stroke patients: effectiveness and safety evaluation
Source: Neurol Sci. 2023 Sep 21;45(1):65–74. doi: 10.1007/s10072-023-07032-z (PMC10761518; doi:10.1007/s10072-023-07032-z)
Supplement: Supplementary file 2 — (DOCX 38.5 kb) [file 10072_2023_7032_MOESM2_ESM.docx]

| **First author** | **Title** | **Stem cell type** | **Stem cell source** | **Administration route** | **Administration window (***following stroke onset***)** | **Outcomes (***serious adverse events and most relevant NIHSS, BI or mRS reports***)** |
| --- | --- | --- | --- | --- | --- | --- |
| Savitz Si et al, 2019 | A Phase 2 Randomized, Sham-Controlled Trial of Internal Carotid Artery Infusion of Autologous Bone Marrow-Derived ALD-401 Cells in Patients with Recent Stable Ischemic Stroke (RECOVER-Stroke) | ALD-401 | autologous | intraarterial | 13-19 days | **Safety*:*** Four patients in the ALD-401 group but none in the control group were found to have small ischemic lesions on MRI at 24 hrs. after the injection. None were associated with worsening of NIHSS or mRS scores. Four subjects in the ALD-401 treatment group and 1 control subject sustained noted seizures following treatment. All were temporally distant from the procedure and considered as not associated. No instances of neoplasia were reported.  **Effectiveness:** No difference in the primary efficacy endpoint (mRS) between the groups after three months. |
| Vahidy FS. et al. 2019 | Intravenous Bone Marrow Mononuclear Cells for Acute Ischemic Stroke: Safety, Feasibility, and Effect Size from a Phase I Clinical Trial | BM-MSCs | autologous | intravenous | 24–72 hours | **Safety*:*** One patient experienced hyperperfusion syndrome following an intra-arterial (IA) therapy procedure and considered as non-related to the study. Two events of hemorrhagic transformation were asymptomatic and without any neurological deterioration.  **Effectiveness:** The day-90 mRS is assessed via telephone interviews and provided as seven point (0–6) ordinal scale. Control group had greater odds for having a higher mRS as compared with the experimental group (OR: 2.72, 95% CI: 1.18–6.30). |
| Levy ML et al. 2019 | Phase I/II Study of Safety and Preliminary Efficacy of Intravenous Allogeneic Mesenchymal Stem Cells in Chronic Stroke | BM-MesSCs | allogeneic | intravenous | >6 months | **Safety** *(primary endpoint):* An event of complex partial epilepsy was reported and considered as unrelated.  **Effectiveness** (*secondary endpoint):* BI increased by 6.8±11.4 points (mean±SD) at 6-months (P=0.002) and by 0.8±15.5 points at 12-months (P<0.001) post-infusion. |
| Fang J. et al. 2019 | Autologous Endothelial Progenitor Cells Transplantation for Acute Ischemic Stroke: A 4-Year Follow-Up Study | EPCs and  BM-MSCs | autologous | intravenous | within 7 days | **Safety** *(primary endpoint):* No event was judged to be attributable to stem cell transplantation. One patient in EPC group had deep venous thrombosis, which was considered as unrelated.  **Effectiveness**: No significant differences were found between the groups at each time point for the mRS, BI and NIHSS. |
| Bhatia V. et al. 2018 | Randomized Assessment of the Safety and Efficacy of Intra-Arterial Infusion of Autologous Stem Cells in Subacute Ischemic Stroke. | BM-MSCs | autologous | intraarterial | 8-15 days | **Safety** *(primary endpoint):* No procedure-related mortality, complication, new infarct, or symptomatic intracranial hemorrhage is reported. Although one patient in the intervention group had an episode of infarction on the contralateral side 2.5 months after the procedure. No evidence of neoplasm has been reported.  **Effectiveness** *(secondary endpoint: mRS at 6 months):* 8 (80 %) patients in the intervention group and 4 (40%) in the control group achieved good outcome (P = .068; odds ratio=6; 95% CI odds ratio, 0.81– 44.31). |
| Laskowitz DT et al. 2018 | Allogeneic Umbilical Cord Blood Infusion for Adults with Ischemic Stroke: Clinical Outcomes from a Phase I Safety Study | Umbilical cord blood MesSCs | allogenic | intravenous | 3-10 days | **Safety** *(primary endpoint):* No events that were determined to be definitely or probably related to investigational treatment, only one case of pruritis of moderate severity was determined to be possibly related to the investigational treatment.  **Effectiveness** *(secondary endpoint: mRS at 3 months):* Patients improved at least one grade in mRS relative to baseline. |
| Deng L. et al 2018 | Intrathecal Injection of Allogenic Bone Marrow-Derived Mesenchymal Stromal Cells in Treatment of Patients with Severe Ischemic Stroke: Study Protocol for a Randomized Controlled Observer-Blinded Trial. | BM-MesSCs | autologous | intrathecal | between 30 and 90 days | Not applicable (ongoing trial AND/OR not enough data) |
| Osanai T. et al. 2017 | Treatment evaluation of acute stroke for using in regenerative cell elements (TREASURE) trial: Rationale and design. | MultiStem (HLM051) | allogenic | intravenous | 18 -36 hours | Not applicable (ongoing trial AND/OR not enough data) |
| Tsang KS. et al. 2017 | Phase I/II randomized controlled trial of autologous bone marrow-derived mesenchymal stem cell therapy for chronic stroke. | BM-MesSCs | autologus | intravenous | <1 year | **Safety:** No serious adverse events have been reported.  **Effectiveness*:*** mBI had a tendency to improve at 12th, 16th, 24th, 36th and 60th weeks upon completion of the treatment (no control arm data available). |
| Hess David C. et al. 2017 | Safety and efficacy of multipotent adult progenitor cells in acute ischaemic stroke (MASTERS): a randomised, double-blind, placebo-controlled, phase 2 trial | M-APCs | allogenic | intravenous | 24 - 48 hours | **Safety:** Life-threatening adverse events and death were reported to be not significantly different between the arms.  **Effectiveness**: No difference between the multipotent adult progenitor cell group and placebo groups in global  stroke recovery at day 90 (odds ratio 1·08 [95% CI 0·55–2·09], p=0·83). |
| Shichinohe H. et al. 2017 | Research on advanced intervention using novel bone marrOW stem cell (RAINBOW): a study protocol for a phase I, open-label, uncontrolled, dose-response trial of autologous bone marrow stromal cell transplantation in patients with acute ischemic stroke | BM-MesSCs | autologus | intracerebral | less than 74 days | **Safety** *(primary endpoint):* No serious adverse event has been reported.  **Effectiveness**: 5 out of 6 patients exerted favorable motor recoveries. |
| Steinberg Gary K. et al. 2016 | Clinical Outcomes of Transplanted Modified Bone Marrow–Derived Mesenchymal Stem Cells in Stroke | BM-MesSCs | allogenic | intracerebral | between 6 and 60 months | **Safety** *(12 months period):* A single patient developed an asymptomatic subdural fluid collection that was definitely related to the procedure and was resolved by surgical intervention. 2 cases of convulsion (one is considered as surgery related), 1 case of transient ischemic attack and 2 of subdural hematoma were considered as non-related. All serious events were resolved without sequelae.  **Effectiveness*:*** NIHSS: mean decrease 2.00 (95% confidence interval, −2.7 to −1.3; P<0.001). No changes in mRS score. |
| Kalladka D. et al. 2016 | Human neural stem cells in patients with chronic ischaemic stroke (PISCES): a phase 1, first-in-man study. | CTX-DP immortalised human neural stem-cell line | allogenic | intracerebral | between 6 and 60 months | **Safety** (*primary endpoint for > 6 months*): 1 extradural and 1 subdural hematoma cases were considered as surgery related. 1 case of seizure considered as not related. 1 case of occipital infarction as attributed to withholding antiplatelet therapy before surgery. A superficial malignant melanoma occurred in one patient with a history of chronic sun exposure.  **Effectiveness*:*** Improvements are reported over time in NIHSS and BI scores. Disability was unchanged in seven of 11 patients. At 24 months, disability was unchanged in seven patients, worsened by two grades in one, and improved by one grade in three. |
| Ghali AA et al. 2016 | Intra-arterial Infusion of Autologous Bone Marrow Mononuclear Stem Cells in Subacute Ischemic Stroke Patients. | BM-MSCs | autologus | intraarterial | 1 week and up to 3 months | **Safety**: 1 of the patients died due to severe chest infection 5 months post-procedure. No report of seizures, tumor formation or recurrent stroke in the treatment group.  **Effectivenes*s****:* mRS, NIHSS and BI improved over time, but there was no significant difference between the study arms. |
| Bhasin A. et al.,2016 | Paracrine Mechanisms of Intravenous Bone Marrow-Derived Mononuclear Stem Cells in Chronic Ischemic Stroke. | BM-MSCs | autologus | intravenous | 3 months to 1.5 years | **Safety** *(primary endpoint):* No early or late adverse reactions during and after transplantation were reported until 8 weeks.  **Effectiveness***:* No statistically significant difference was observed in mBI score  (95% CI 14.3–4.5, p = 0.31) at 2 months. |
| Taguchi A. et al. 2015 | Intravenous Autologous Bone Marrow Mononuclear Cell Transplantation for Stroke: Phase1/2a Clinical Trial in a Homogeneous Group of Stroke Patients | BM-MSCs | autologous | intravenous | 7-10 days | **Safety**: One case of aspiration pneumonia and sepsis 3 months after, considered as non-related, one case of recurrent stroke, considered as unclear.  **Effectiveness**: NIHSS at day 30 (primary efficacy outcome measure): Mean improvement of 4.8 ± 4.6 (p<0.01, 95% confidence intervals [1.805, 7.694]). |
| Prasad K. et al. 2014 | Intravenous autologous bone marrow mononuclear stem cell therapy for ischemic stroke: a multicentric, randomized trial | BM-MSCs | autologous | intravenous | 7- 30 days | **Safety**: 1 case of serious deterioration in treatment group has been recorded. Life-threatening adverse events and death were reported to be not significantly different between the arms.  **Effectiveness** (at day 180): No significant difference between arms BI (63.1 versus 63.6; P=0.92), mRS (P=0.53) >3 (47.5% versus 49.2%; P=0.85), and NIHSS scores (6.3 versus 7.0; P=0.53) |
| Qiao LY et al. 2014 | A two-year follow-up study of cotransplantation with neural stem/progenitor cells and mesenchymal stromal cells in ischemic stroke patients | cotransplantation of NSPCs and BM-MesSCs  and injection of only BM-MesSCs | autologous | intravenous and intracerebroventricular | <1 week for one patient, 1 week  to 1 month for three patients, and during stroke  sequelae for two patients (0.5–2 years) | **Safety**: Single patient exerted temporary dizziness. No tumorigenesis at 2 years follow-up.  **Effectiveness**: Five cases exhibited decreases in NIHSS scores of more than two points 3 months after the procedure. |
| Banerjee S. et al. 2014 | Intra-Arterial Immunoselected CD34+ Stem Cells for Acute Ischemic Stroke | CD34+ hematopoietic stem/progenitor cells | autologous | intraarterial | within 7 days | **Safety** *(primary endpoint):* No serious adverse events have been reported.  **Effectiveness**: Significant improvement was seen in the mean NIHSS score, from 10.40 to 2.20 (95% confidence interval [CI] 3.69–12.71; p =  .007), and mean mRS, from 3.80 to 1.60 (95% CI 1.64–2.76; p =.0004), from day 0 to day 180. |
| Chen DC et al. 2014 | Intracerebral implantation of autologous peripheral blood stem cells in stroke patients: a randomized phase II study | CD34^+^ PBSCs | autologous | intracerebral | 6 months to 5 years following stroke onset | **Safety:** No serious adverse events have been reported.  **Effectiveness**: mRS and NIHSS scores showed a significant time-dependent improvement compared to control group during the 12 months follow-up. |
| Jiang Y. et al. 2013 | Feasibility of delivering mesenchymal stem cells via catheter to the proximal end of the lesion artery in patients with stroke in the territory of the middle cerebral artery | umbilical cord MesSCs | allogeneic | intraarterial | within 3 months | **Safety**: No serious adverse events have been reported.  **Effectiveness**: mRS improved in two patients, evaluated at 90 and 180 days after stem cell therapy. There was no significant improvement in case of haemorrhagic stroke patient. |
| Chen L. et al. 2013 | Multiple cell transplantation based on an intraparenchymal approach for patients with chronic phase stroke | OECs, NPCs, SCs, umbilical cord MesSCs | allogeneic | intracranial parenchymal, intrathecal implantation, intravenous | 6 months to 20 years | **Safety*:*** No serious adverse events have been reported.  **Effectiveness***:* All patients achieved some degree of neurological function amelioration, including improvements in speech, muscle power, muscular tension, balance, pain relief, and respiratory ability. The BI increased from preoperative 59.50 ± 28.52 to postoperative 64.00 ± 29.70 (p = 0.041). |
| Prasad K. et al. 2012 | Autologous intravenous bone marrow mononuclear cell therapy for patients with subacute ischaemic stroke: a pilot study. | BM-MSCs | autologus | intravenous | 7-30 days | **Safety*:*** One patient developed small infarct which was clinically silent.  **Effectiveness***:* Favourable outcome (defined as mRS score of 2 or less or BI score of 75 to 100 at six months after stem cell therapy.) was found in 7 of 11  (64%): as per scales- BI 7 of 11 (64%), NIHSS 5 of 10 (50%) and mRS 6 of 11 (54.5%). |
| Bhasin A. et al. 2012 | Autologous intravenous mononuclear stem cell therapy in chronic ischemic stroke. | BM-MSCs and BM-MesSCs | autologus | intravenous | 3 months to 2 years | **Safety*:*** No serious adverse events have been reported.  **Effectiveness***:* Only mBI showed statistically significant improvement at 24 weeks (p<0.05) and only in BM-MSCs’ group. |
| Honmou O. et al. 2011 | Intravenous administration of auto serum-expanded autologous mesenchymal stem cells in stroke | BM-MesSCs | autologous | intravenous | 36–133 days | **Safety:** No serious adverse events have been reported.  **Effectiveness**: The median daily rate of NIHSS change was 0.36 during the first week post-infusion, compared with a median daily rate of change of 0.04 from the first day of testing to immediately before infusion. The mRS change showed similar trend. |
| Lee JS et al. 2010 | A long-term follow-up study of intravenous autologous mesenchymal stem cell transplantation in patients with ischemic stroke | BM-MesSCs | autologous | intravenous | <7 weeks | **Safety:** 21 patients in control and 4 in experimental group died during the follow up period. No case of malignant tumor has been reported. 5 in control and 3 in experimental had seizures after stroke. 3 and 4 patients developed vascular recurrent stroke in control and experimental groups respectively.  **Effectiveness**: *(~3.2 years follow up)* In the experimental group, 11 of 16 patients had a negative rank, whereas only four patients had a positive rank. There tended to be more patients with improved outcome than with worsened outcome in the treatment group. The proportion of patients with mRS 0–3 increased in the treatment group but not in the control group. |
| Bang OY et al. 2005 | Autologous mesenchymal stem cell transplantation in stroke patients. | BM-MesSCs | autologous | intravenous | <7 weeks | **Safety:** No serious adverse events have been reported for the 1-year follow up period.  **Effectiveness**: Outcomes improved in treated patients compared with the control patients: the BI and mRS. The NIHSS change was less prominent. |
| Suarez-Monteagudo C et al. 2009 | Autologous bone marrow stem cell neurotransplantation in stroke patients. An open study | BM-MSCs | autologous | intracerebral | 1-10 years | **Safety*:*** No serious adverse events have been reported for the 1-year follow up period.  **Effectiveness**: The patients showed a reduced motor defect and spasticity, an increased functional capacity,  better neurological condition, equilibrium and locomotion. |
| Rabinovich SS et al. 2005 | Cell therapy of brain stroke. | IN and HHCs | allogeneic | subarachnoid (spinal) | 4-24 months | **Safety:** No serious adverse events have been reported. **Effectiveness**: 6 months after cell therapy functional activity significantly increased in contrast to clinically comparable control group. |
| Kondziolka D. et al. 2000 | Transplantation of cultured human neuronal cells for patients with stroke | LBS-neurons | allogeneic | intracerebral | 6 months to 6 years | **Safety:** 1 patient had seizure 6 months after surgery. 1 brainstem stroke 24 weeks after cell implantation. No deaths are reported.  **Effectiveness**: At the 24-week follow-up  evaluation post implantation of LBS-neurons, eight patients had improved scores on the NIHSS (range, 21 to 24 points), one patient was unchanged, and three patients deteriorated (range, 1 to 2 points) compared with their baseline scores. At baseline the mean BI scores were 77.5 for the 2 million group and 73.8 for the 6 million group. At week 24 the mean BI score dropped to 72.1 for the 2 million group and increased to 80.0 for the 6 million group. |
